# Efficacy and safety of sequential immunosuppressive treatment for severe IgA nephropathy: A retrospective study

**DOI:** 10.3389/fphar.2023.1093442

**Published:** 2023-03-14

**Authors:** Mian-Na Luo, Qingjun Pan, Ting Ye, Shangmei Li, Lawei Yang, Hua-Feng Liu, Yongzhi Xu

**Affiliations:** ^1^ Guangdong Provincial Key Laboratory of Autophagy and Major Chronic Non-Communicable Diseases, Affiliated Hospital of Guangdong Medical University, Zhanjiang, China; ^2^ Department of Nephrology, Affiliated Hospital of Guangdong Medical University, Zhanjiang, China

**Keywords:** severe IgA nephropathy, progressive IgA nephropathy, sequential immunosuppressive therapy, efficacy analysis, retrospective study

## Abstract

**Background:** This study compared the efficacy and safety of sequential immunosuppressive therapy in patients with non-end-stage IgA nephropathy (IgAN) with Lee’s classification of IV ∼ V and provided evidence for the use of immunotherapy in patients with severe IgAN.

**Methods:** We retrospectively analyzed the clinical data of patients with Lee’s IV ∼ V non-end-stage IgA nephropathy.

**Results:** 436 patients were diagnosed with IgAN, and 98 patients who met the inclusion criteria were included in this retrospective study. Of these, 17 were in the supportive care group, 20 in the P group (prednisone-only), 35 in P + CTX group (the prednisone combined with cyclophosphamide followed by mycophenolate mofetil), and 26 in the P + MMF group (prednisone combined with mycophenolate mofetil). The four groups showed differences in the segmental glomerulosclerosis score and the proportion of patients with Lee’s grade IV (*p* < 0.05), but no differences in other indicators. Compared with the baseline values, urine protein-to-creatinine ratio (PCR) significantly decreased and serum albumin increased (*p* < 0.05), but there was no significant difference between the groups. The estimated Glomerular Filtration Rate (eGFR) of the P, P + MMF, and P + CTX groups were higher than that of the supportive care group at the 6th and 24th month after treatment (all *p* < 0.05). At the 24th month, the eGFR in the P + CTX group was higher than that in the P + MMF group (*p* < 0.05). The effective remission rate of the P + CTX group was higher than that of the supportive care group (*p* < 0.05). At 12 months, the effective remission rate of the P group was higher than that of the supportive care group (*p* < 0.05). At the 24th month, there was no significant difference in the effective remission rates among the three groups (P, P + MMF, and P + CTX). Nine patients with severe IgA nephropathy reached the endpoint.

**Conclusion:** This study showed that immunosuppressive therapy insevere IgAN patient scan effectively reduce urinary protein, increase albumin, and protect renal function in the early stages of IgAN. P + CTX is the most commonly used, which has a high effective remission rate of urine protein and a low incidence of end-point events.

## Introduction

Immunoglobulin A nephropathy (IgAN) is the most common primary glomerulonephritis worldwide. The most important pathological feature is the deposition of a large amount of IgA in the mesangial area of the glomerulus. Its onset is insidious. Approximately 15%–20% of patients will develop end-stage renal disease (ESRD) within 10 years, and the proportion of patients who develop ESRD within 20 years can reach as high as 40% (Rychlik et al., 1999).

It is well known that IgAN is an autoimmune nephropathy, and the specific pathogenesis has not been elucidated. Galactose-deficient IgA1 (Gd-IgA1) has been reported to be significantly increased in patients with IgAN, and pathogenic immune complexes formed by combining autoantibodies accumulate abnormally in the kidneys, thus activating immune responses (Suzuki et al., 2011). However, neither inhibitory therapy for pathogenic Gd-IgA1(Fellström et al., 2017; Muto et al., 2017) nor biological targeting therapy for immune complex formation (Lafayette et al., 2017) can prove its efficacy and safety. Therefore, reducing the formation of immune complexes and the immune inflammatory response is still the focus of doctors when choosing a treatment plan.

The Kidney Disease Improving Global Outcomes (KDIGO) guidelines recommend that glucocorticoids be used only in patients with advanced high-risk IgAN whose urine protein remains greater than 1g/24 h despite conservative treatment (Rovin et al., 2021). However, even if the urine protein is less than 1 g/24h, the use of corticosteroids can reduce the risk of progression to ESRD in patients (Moriyama et al., 2020), furthermore corticosteroids can improve renal function in patients during long-term follow-up. In contrast, some patients with severe IgAN who did not meet the standard of prednisolone use and received conservative treatment had rapidly deteriorated renal function and even directly entered the state of dialysis (Mitsuiki et al., 2007). Recent prospective cohort studies STOP-IgA trial and the TESTING trial suggested that corticosteroids are effective in reducing urinary protein levels. However, there is little evidence of improvement in renal pathology with treatment (Robert et al., 2016; Lv et al., 2017). Therefore, the indications for and efficacy of corticosteroids in the treatment of IgAN with different degrees of pathological damage are unclear.

Mycophenolate mofetil (MMF) can selectively inhibit lymphocyte proliferation, leading to apoptosis of cytotoxic T lymphocytes. The results of repeated renal biopsy studies showed that, pathological statesand mesangial IgA deposition improved after single-agent MMF treatment in IgAN patients. Moreover, prednisolone combined with MMF therapy can reverse acute histological damage and delay the progression of renal failure (Roccatello et al., 2012; Beckwith et al., 2017; Hou et al., 2017). At present, most studies lack specific histological inclusion and exclusion criteria when recruiting patients, there are few studies on the treatment of severe IgAN, and the efficacy of MMF in IgAN is inconclusive. Cyclophosphamide (CTX) mainly acts on B lymphocytes. Studies have shown that corticosteroid combined with CTX can reduce proteinuria in IgAN patients with moderate and severe pathology, delay the progression of renal function, and significantly improve the survival of IgAN patients (Chen et al., 2003). KDIGO guidelines recommend corticosteroids combined with CTX for the treatment of IgAN patients with rapidly declining renal function and a crescent ratio of >50% (Rovin et al., 2021). However, repeated renal biopsy studies in our center also showed that immunosuppressive therapy can reduce the proportion of crescents in the kidneys of some patients with IgAN, while reducing proteinuria, stabilizing renal function, and slowing chronic renal failure in the short term (Luo et al., 2020).

Therefore, for IgAN, especially in those with severe pathology, the selection and efficacy of immunosuppressive therapy are worthy of further study. This study retrospectively analyzed the efficacy of immunotherapy in patients with severe IgAN and adverse reactions during follow-up to provide a basis for the treatment of patients with severe IgAN.

## Materials and methods

### Study design and population

From January 2016 to February 2020, patients were hospitalized in the Department of Nephrology, Affiliated Hospital of Guangdong Medical University, and underwent renal biopsy. The pathological diagnosis was primary IgAN. The following conditions were applied: aged 18–65 years, Lee’s pathological grade IV to V, the number of glomeruli under the microscope was more than 10, the data for the first hospitalization cases were complete, and the outpatient follow-up data were complete at 6, 12, 18, and 24 months after discharge. The exclusion criteria were as follows: ① use of immunosuppressive agents, ② atypical manifestations of IgAN, such as crescentic nephritis, minimal change disease, membranous nephropathy or other nephropathy, transplanted kidney IgAN, ③ concomitant conditions that affect treatment, such as non-solid malignant tumor, pregnancy, severe malnutrition, liver function damage (alanine aminotransferase increased by more than 2 times), etc., ④renal replacement therapy has been performed or eGFR<15 mL/min/1.73 m^2^, and ⑤secondary renal IgA deposition such as disease associated with viral infections, autoimmune diseases, tumors, etc. The study complied with the Helsinki Declaration and was approved by the ethical review committee of the Affiliated Hospital of Guangdong Medical University.

### Clinical data collection

The data for this study were collected from the hospital information system of our hospital, electronic medical record system, inspection report query system, pathology system of the Institute of Kidney Diseases, and Siyuan Chronic Kidney Disease Outpatient Follow-up System. Three kidney disease professionals performed manual data collection and proofreading. Baseline data included hospitalization data at the time of the first renal biopsy. General patient information, including name, sex, age, and clinical data at onset, such as clinical manifestations at onset, duration of disease, systolic blood pressure (SBP), diastolic blood pressure (DBP), and mean arterial pressure (MPA) were collected. Test data at onset, including 24-h urine protein quantification (24-UPro), urine protein-to-creatinine ratio (PCR), serum creatinine (Scr), eGFR, serum uric acid (SUA), serum albumin (ALB), serum cholesterol (CHO), alanine aminotransferase (ALT), serum cystatin C (Cys C), hemoglobin (HGB), and renal pathological typing, including the Lee’s pathological grade and Oxford pathological classification were collected. Taking renal biopsy as the starting point of treatment, the changes in PCR, eGFR, SUA, ALB, and endpoint events at the 6th, 12th, 18th, and 24th months after treatment were recorded during outpatient follow-up.

### Pathological grading of IgA nephropathy

Lee’s pathological classification is one of the classic pathological classification criteria of IgAN, which has been proved to be effective in predicting the prognosis of IgAN in clinical practice (Frimat et al., 1997), and Lee’s pathological grading elements mainly include glomerular and tubular lesions, including mesangial cell proliferation, mesangial area proliferation, glomerulosclerosis, crescent, tubular lesions and interstitial inflammatory cell infiltration (Lee, 1997). The Oxford Classification includes these 5 parameters, the MEST-C scores. The five features were mesangial hypercellularity (M), segmental glomerulosclerosis (S), tubular atrophy/interstitial fibrosis (T), endocapillary hypercellularity (E) and crescents (C) (Trimarchi et al., 2017).

### Interventions

The patients were divided into a supportive treatment group and three immunosuppressive treatment groups: prednisone-only (P group), prednisone combined with CTX followed by mycophenolate mofetil (P + CTX group), and prednisone combined with MMF (P + MMF group). In P group, the initial dose of prednisone was 0.5–1 mg/kg/d (low-dose prednisone is less than 0.5 mg/kg/d), and it decreased by 10–20% after 2–3 months. The dosage was maintained at 2.5–10 mg/d. CTX was induced by intravenous infusion of 0.5–1.0 g/m^2^ every month for 3–6 months. The therapeutic dose of MMF was 1.0–1.5 g/d, administered orally in two doses. The P + CTX group received sequential MMF maintenance therapy after CTX. The supportive care group mainly received RASS blockers. Specifically, refer to our previous study (Luo et al., 2014; Luo et al., 2020), we advocated personalized administration for each patient’s treatment plan, that is, combining the patient’s urine protein (whether it is greater than 1 g/d), histological lesions (whether there are active pathological changes, such as crescent, inflammatory cell infiltration, etc.) and the patient’s informed consent (informing the drug of possible side effects and drug prices). Based on the above consideration, a plan was formulated for each patient. At the same time, the patients were followed up regularly in the outpatient department, and the medication will be adjusted according to the problems in the follow-up process. General and serious adverse reactions during the follow-up period were recorded. Severe adverse reactions refer to reactions that are fatal, disabling, or lead to prolonged hospitalization. Time of occurrence, severity, treatment plan, and outcomes were recorded. Adverse reactions included drug-induced hepatitis, elevated blood sugar, femoral head necrosis, drug-induced menopause, menopause, and premature menopause. Symptoms of various systemic infections of the digestive, respiratory, and urinary systems, blood, and skin were included. The termination date of the study was February 2022.

### Definitions and calculations

1) Mean arterial pressure (MAP, mmHg): MAP = diastolic blood pressure + 1/3 (systolic blood pressure—diastolic blood pressure), 2) course of disease: the time from the first onset to the first renal biopsy, calculated in months, 3) severe IgAN, Lee’s pathological grade IV ∼ V primary IgAN, and 4) eGFR was calculated according to the MDRD formula.

### Efficacy evaluation

Efficacy evaluation: ①Complete remission:24-UPro≤300 mg or PCR≤0.3 g/g, and Scr increased by <15% compared with the baseline, ②Partial remission:24-UPro>300 mg or PCR>0.3 g/g, but decreased by >50% compared with the baseline, and Scr was increased by <15% compared with the baseline, and ③Invalid: the above two criteria were not met. Effective remission = (complete remission + partial remission)/total number of cases × 100%.

Endpoint events: doubling of serum creatinine level, entering dialysis, or reaching ESRD.

Relapse: After complete or partial remission, urine protein measurement ≥1.0g/24 h two consecutive times.

### Statistical analysis

SPSS 26.0 statistical analysis software (IBM Corporation, Armonk, NY, USA) was used to organize, count, and analyze the data. The Shapiro–Wilk test was used to test the normality of the measurement data. The normal distribution was described by Mean±s.d, while the non-normal distribution was described by M(P25-P75) and the enumeration data were described by [n (%)]. Normally distributed indicators among multiple groups were compared using variance analysis, and the S-N-K method was used for pairwise comparisons. Non-normally distributed indicators among multiple groups were compared using the Kruskal–Wallis H test. The rates between groups were compared using the chi-square test. *p* < 0.05 was considered statistically significant.

## Results

### Study design and population

**FIGURE 1:**
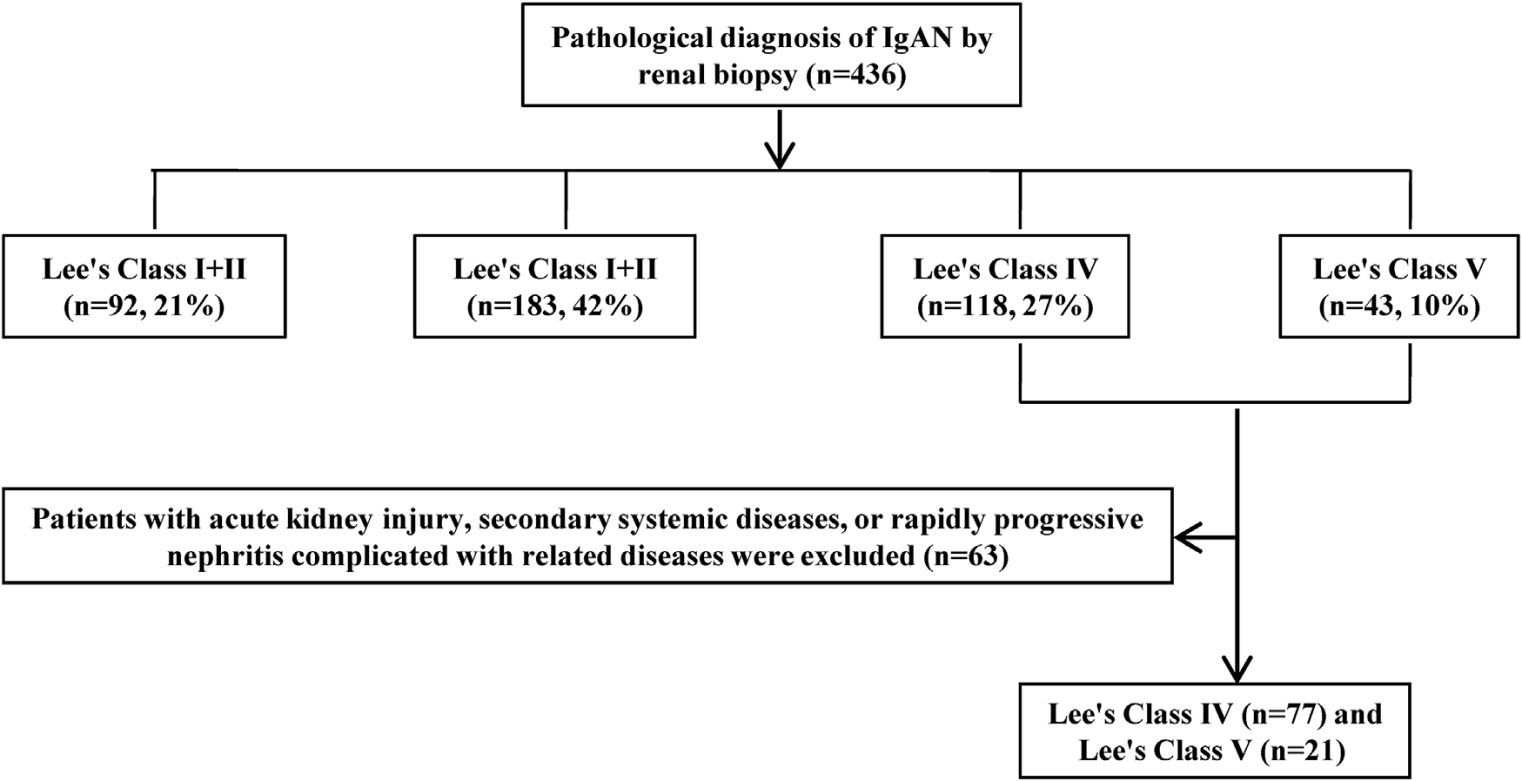
Flow–chart of enrollment and exclusion.

A total of 436 patients with IgAN were diagnosed by renal biopsy at our center between January 2016 and February 2020. The selection process is shown in Figure 1. A total of 98 eligible patients (42 males and 56 females) with an age of 38.1 ± 11.3 years were included. The median disease duration before renal biopsy was 6.5 months. Baseline data of the different treatment groups for severe IgAN is shown in Table 1. The supportive care, P, P + CTX, and P + MMF groups show significant differences in the segmental glomerulosclerosis score (S) and Lee’s IV ratio (*p* < 0.05). There is no significant difference in the general clinical case data, clinical test indexes, and Oxford classification indexes among the four treatment groups (*p* > 0.05).

**TABLE 1:** Comparison of baseline data in different treatment groups for severe IgAN (n = 98).

Group		P + CTX group	P + MMF group	P Group	Supportive treatment group	*P*
		(n = 35)	(n = 26)	(n = 20)	(n = 17)	
men, n (%)		15 (42.90)	11 (42.30)	8 (40.00)	8 (47.10)	0.98
Year at biopsy (Y)		35.5 ± 9.2	39.3 ± 13.0	36.9 ± 12.4	43.2 ± 10.3	0.11
SBP (mmHg)		134.7 ± 19.1	134.4 ± 23.8	135.8 ± 34.1	134.6 ± 22.7	1.00
DBP (mmHg)		83.0 ± 14.2	80.8 ± 13.2	82.0 ± 15.7	81.4 ± 12.7	0.94
MAP (mmHg)		100.2 ± 15.1	98.7 ± 16.0	99.9 ± 20.3	99.1 ± 14.8	0.99
Hematuria, n (%),		25 (71.40)	19 (73.10)	15 (75.00)	9 (52.90)	0.44
HGB (g/L)		129.57 ± 21.38	126.25 ± 17.35	122.09 ± 21.70	129.06 ± 20.61	0.59
ALT (U/L)		13.60 (8.50–22.90)	14.70 (9.88–20.10)	16.25 (10.80–25.15)	18.00 (14.00–26.65)	0.41
CHO (mmol/L)		5.42 ± 1.32	5.54 ± 0.93	5.26 ± 1.32	5.58 ± 1.35	0.84
Cys C (mg/L)		0.91 (0.69–1.29)	0.95 (0.75–1.29)	0.99 (0.70–1.13)	1.13 (0.75–1.33)	0.62
SUA (μmol/L)		403.30 (358.60–488.50)	413.05 (352.95–523.25)	441.35 (360.00–555.67)	504.00 (426.90–552.50)	0.13
BUN (mmol/L)		5.00 (3.75–8.28)	6.21 (4.81–9.27)	6.40 (4.31–7.89)	8.60 (6.19–10.86)	0.12
ALB (g/L)		38.80 (34.00–42.40)	37.05 (34.63–42.15)	36.65 (33.20–41.33)	39.90 (35.10–43.70)	0.63
Scr (μmol/L)		94.00 (64.00–153.00)	100.00 (87.00–142.25)	97.00 (76.00–119.50)	136.00 (88.50–209.50)	0.17
PCR (g/g)		1.71 (0.85–3.02)	1.35 (0.83–2.00)	1.17 (0.63–2.71)	0.84 (0.45–2.88)	0.49
24-UPro (g/d)		1.49 (1.03–2.92)	1.79 (0.82–2.52)	1.25 (0.76–3.37)	1.14 (0.61–3.39)	0.47
eGFR (mL/min/1.73m^2^)		75.77 (43.11–116.19)	64.66 (38.27–86.68)	70.79 (60.13–98.29)	42.61 (27.46–80.82)	0.14
CKD staging (%)						0.24
	1	16 (45.70)	6 (23.10)	5 (25.00)	3 (17.60)	
	2	6 (17.10)	9 (34.60)	9 (45.00)	3 (17.60)	
	3	8 (22.90)	6 (23.10)	4 (20.00)	6 (35.30)	
	4	5 (14.30)	5 (19.20)	2 (10.00)	5 (29.40)	
*Lee’s grades, n (%)		31(88.60)	21(80.80)	17(85.00)	8(47.10)	0.01
M1, n (%)		19 (54.30)	15 (57.70)	13 (65.00)	5 (29.40)	0.16
*S1, n (%)		35 (100.00)	26 (100.00)	20 (100.00)	13 (76.50)	0.01
E1, n (%)		16 (45.70)	15 (57.70)	11 (55.00)	5 (29.40)	0.28
T (1 + 2), n (%)		28 (80.00)	23 (88.50)	17 (85.00)	16 (94.10)	0.63

*Comparison among four groups, *p* < 0.05.

### Treatment options for severe IgAN

Atotal of 94 patients (95.92%) in the four groups used renin-angiotensin system inhibitors (RASI), and the baseline Scr of four patients without RASI was >265 μmol/L, including one in the supportive treatment group, two in the P + MMF group, and one in the P group. The total dose of CTX was 3.60 (2.40–4.80) g, of which 16 patients (45.71%) had a total dose greater than 3.6 g. The initial doses of prednisone in the P, P + MMF, and P + CTX group were 39.47 ± 12.12 mg/d, 34.8 ± 11.50 mg/d and 35.94 ± 11.80 mg/d, respectively. There is no significant difference among the three groups. The total doses of prednisone were 9.28 ± 2.17 g, 9.03 ± 1.42 g, and 9.44 ± 1.76 g, respectively, and there is no significant difference among the three groups (see Table 2).

**TABLE 2:** Prednisone treatment plan.

Group	P Group	P + MMF group	P + CTX group
Initial prednisone dosage (mg/d)	39.47 ± 12.12	34.80 ± 11.50	35.94 ± 11.80
Total prednisone (g)	9.28 ± 2.17	9.03 ± 1.42	9.44 ± 1.76

### Follow-up indicators in different treatment groups for severe IgAN

#### Urine protein/creatinine ratio (PCR)

A sshown in Table 3, at 6, 12, 18, and 24 months of treatment, the PCR of each group decrease compared with the baseline value, and the difference was statistically significant (*p* < 0.05). There is no significant difference in the PCR among the four groups (*p* > 0.05).

**TABLE 3:** 24 h-UPro comparison of different treatment groups in severe IgAN during follow-up.

Group	P + CTX group	P + MMF group	P Group	Supportive treatment group
	(n = 35)	(n = 26)	(n = 20)	(n = 17)
Baseline	1.49 (1.03–2.92)	1.79 (0.82–2.52)	1.25 (0.76–3.37)	1.14 (0.61–3.39)
The 6th month	0.40 (0.16–1.57)*	0.35 (0.12–0.88)*	0.44 (0.31–1.16)*	0.42 (0.18–1.68)*
The 12th month	0.35 (0.16–1.04)*	0.26 (0.08–0.97)*	0.43 (0.09–0.90)*	0.37 (0.32–1.19)*
The 18th month	0.27 (0.14–0.74)*	0.18 (0.13–0.56)*	0.27 (0.15–0.54)*	0.55 (0.20–0.85)*
The 24th month	0.25 (0.17–0.45)*	0.24 (0.12–0.74)*	0.33 (0.16–0.82)*	0.45 (0.10–0.83)*

*Compared with baseline, *p* < 0.05.

#### Serum albumin (ALB)

As shown in Table 4, at 6, 12, 18, and 24 months of treatment, the ALB of the four groups is higher than the baseline values (*p* < 0.05). There is no statistically significant difference in ALB between the groups.

**TABLE 4:** Comparison of ALB in different treatment groups of severe IgAN.

Group	P + CTX group (n = 35)	P + MMF group (n = 26)	P Group (n = 20)	Supportive treatment group (n = 17)
Baseline	38.80 (34.00–42.40)	37.05 (34.63–42.15)	36.65 (33.20–41.33)	39.90 (35.10–43.70)
The 6th month	43.30 (42.20–46.00)*	42.75 (39.92–46.73)*	42.70 (39.78–46.13)*	42.90 (39.50–46.40)*
The 12th month	43.50 (41.00–46.00)*	44.75 (40.65–46.10)*	44.20 (42.60–45.80)*	44.30 (42.00–45.00)*
The 18th month	44.60 (42.50–47.70)*	45.00 (41.50–46.98)*	45.25 (42.75–46.80)*	44.30 (43.30–46.60)*^,^#
The 24th month	45.00 (43.80–47.70)*^,#^	44.90 (41.65–46.90)*	44.70 (42.93–46.83)*	43.30 (42.20–43.90)*^,#,&^

*Compared with baseline, *p* < 0.05.

#Compared with 6 months, *p* < 0.05.

& Compared with 12 months, *p* < 0.05.

#### Glomerular filtration rate (eGFR)

As shown in Table 5, the eGFR of the four groups remain stable or increased from the baseline during the treatment period. Comparing the immunosuppressant and supportive care groups, the eGFR of the P + CTX, P + MMF, and P groups is higher than that of the supportive care group at 6 and 24 months of treatment (*p* < 0.05). Among them, the eGFR of the P and P + CTX groups at the 12th month of follow-up is higher than that of the supportive care group (*p* < 0.05). Comparing immunosuppressant groups, at 6 months, 12 months, and 24 months of treatment, the eGFR in the P + CTX group is higher than that in the P + MMF group (*p* < 0.05). The differences are not statistically significant.

**TABLE 5:** Comparison of eGFR in different treatment groups of severe IgAN.

Group	P + CTX group (n = 35)	P + MMF group (n = 26)	P Group (n = 20)	Supportive treatment group (n = 17)
Baseline	75.77 (43.11–116.19)	64.66 (38.27–86.68)	70.79 (60.13–98.29)	42.61 (27.46–80.82)
The 6th month	75.27 (53.04–99.36)*	62.87 (47.82–80.84)*^,#^	71.13 (55.61–87.33)*	45.45 (28.45–68.95)
The 12th month	72.57 (54.56–105.46)*	59.79 (49.01–82.83)^#^	75.04 (55.09–107.48)*	51.16 (27.83–70.86)
The 18th month	74.37 (54.06–105.84)	69.64 (48.87–85.04)	78.80 (56.50–105.84)	52.20 (31.26–75.25)
The 24th month	81.68 (56.01–112.74)*	62.29 (50.11–84.71)*,^#^	79.06 (50.15–107.25)*	46.79 (26.90–71.00)

*Compared with supportive care group, *p* < 0.05.

#Compared with P + CTX group, *p* < 0.05.

#### Serum uric acid (SUA)

As shown in Table 6, the SUA of the P + MMF, P, and supportive care groups shows a downward trend with the prolongation of treatment time, whereas the SUA of the P + CTX group shows an upward trend at 18 and 24 months of treatment. The SUA levels in the supportive care group and the P group are lower than the baseline value in the 12th month of treatment (*p* < 0.05). At 6, 12, 18, and 24 months of treatment, SUA levels in the P + CTX, P + MMF, and P groups are lower than those in the supportive care group (*p* > 0.05).

**TABLE 6:** Comparison of SUA in different treatment groups of severe IgAN.

Group	P + CTX group (n = 35)	P + MMF group (n = 26)	P Group (n = 20)	Supportive treatment group (n = 17)
Baseline	403.30 (358.60–488.50)	413.05 (352.95–523.25)	441.35 (360.00–555.67)	504.00 (426.90–552.50)
The 6th month	389.00 (330.30–494.30)	382.30 (338.10–415.78)	437.50 (348.50–481.75)*	446.00 (387.00–519.25)
The 12th month	386.00 (313.70–498.00)	390.75 (345.17–412.33)	406.00 (345.00–476.00)*	447.00 (334.00–518.00)*
The 18th month	412.90 (350.00–537.00)	390.00 (350.58–430.90)	405.00 (325.20–465.00)	456.50 (411.10–517.30)
The 24th month	432.65 (336.42–506.65)	391.65 (339.30–459.10)	428.75 (285.48–513.40)	430.60 (324.00–504.00)

*Compared with baseline, *p* < 0.05.

### Curative effect of different treatment groups for severe IgAN

As shown in Table 7, the effective remission rate in the P + CTX group shows an upward trend with prolongation of treatment time, while the effective remission rate in the supportive care group shows a downward trend. At the 12th, 18th, and 24th months of treatment, the effective remission rate in the P + CTX group is higher than that in the supportive care group (*p* < 0.05). In addition, after 12 months of treatment, the effective remission rate in the P group is higher than that in the supportive care group (*p* < 0.05). There is no significant difference in the effective remission rates among the three immunotherapy groups.

**TABLE 7:** Comparison of remission in different treatment groups for severe IgAN.

Treatment time	Efficacy evaluation	P + CTX group (n = 35)	P + MMF group (n = 26)	P Group (n = 20)	Supportive treatment group (n = 17)
The 6th month	Complete remission, n (%)	16 (45.70)	12 (46.20)	4 (20.00)	5 (29.40)
	Partial remission, n (%)	8 (22.90)	5 (19.20)	8 (40.00)	2 (11.80)
	Invalid, n (%)	11 (31.40)	9 (34.60)	8 (40.00)	10 (58.80)
	Effective remission rate (%)	68.60	65.40	60.00	41.20
The 12th month	Complete remission, n (%)	17 (48.60)	12 (46.20)	8 (40.00)	2 (11.80)
	Partial remission, n (%)	11 (31.40)	3 (11.50)	7 (35.00)	2 (11.80)
	Invalid, n (%)	7 (20.00)	11 (42.30)	5 (25.00)	13 (76.50)
	Effective remission rate (%)	80.00*	57.70	75.00*	23.60
The 18th month	Complete remission, n (%)	19 (54.30)	13 (50)	9 (45.00)	2 (11.80)
	Partial remission, n (%)	9 (25.70)	4 (15.40)	4 (20.00)	3 (17.60)
	Invalid, n (%)	7 (20.00)	9 (34.60)	7 (35.00)	12 (70.60)
	Effective remission rate (%)	80.00*	65.40	65.00	29.40
The 24th month	Complete remission, n (%)	17 (48.60)	12 (46.20)	9 (45.00)	3 (17.60)
	Partial remission, n (%)	13 (37.10)	4 (15.40)	4 (20.00)	2 (11.80)
	Invalid, n (%)	5 (14.30)	10 (38.50)	7 (35.00)	12 (70.60)
	Effective remission rate (%)	85.70*	61.60	65.00	29.40

*Compared with supportive care group, *p* < 0.05.

### Endpoint events in different treatment groups for severe IgAN

**FIGURE 2:**
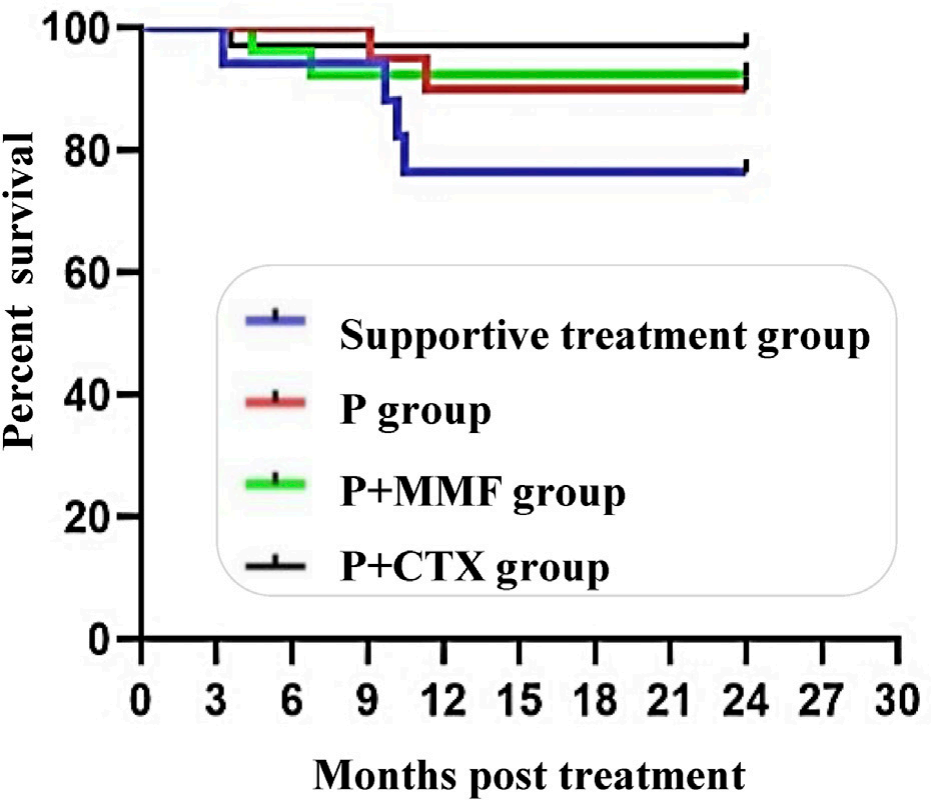
Survival curves of in different treament groups for severe IgAN.

Nine patients reached the endpoint, including four patients (23.50%) in the supportive care group, two patients (10.00%) in the P group, one patient (2.90%) in the P + CTX group, and two patients (7.70%) in the P + MMF group. There is no significant difference in the incidence of end-point events among the four groups, and the survival curve is shown in Figure 2.

### Adverse reactions in different treatment groups of severe IgAN

As shown in Table 8, adverse reactions occurred in 18 patients and the number of cases was 20. No drug-related adverse reactions were observed in the supportive care group, whereas adverse reactions occurred in the P, P + MMF, and P + CTX groups. The rates are 25.00% (5/20), 19.20% (5/26) and 22.90% (8/35), respectively. The incidence of adverse reactions in the P group is higher than that in the P + CTX and P + MMF groups (*p* > 0.05), and the incidence of adverse reactions in the P + CTX group is higher than that in the P + MMF group (*p* > 0.05).

**TABLE 8:** Comparison of adverse reactions in different treatment groups of severe IgAN.

Adverse reactions	P + CTX group (n = 35)	P + MMF group (n = 26)	P Group (n = 20)	Supportive treatment group (n = 17)
Urinary tract infection, n (%)	2 (5.70)	1 (3.80)	1 (5.00)	0 (0.00)
Respiratory infection, n (%)	5 (14.30)	2 (7.60)	4 (20.00)	0 (0.00)
Gastrointestinal bleeding, n (%)	1 (2.90)	1 (3.80)	0 (0.00)	0 (0.00)
Tumor, n (%)	0 (0.00)	0 (0.00)	0 (0.00)	0 (0.00)
Shingles, n (%)	0 (0.00)	0 (0.00)	2 (10.00)	0 (0.00)
Abnormal liver function, n (%)	0 (0.00)	1 (3.80)	0 (0.00)	0 (0.00)
Total, n (%)	8 (22.90)	5 (19.20)	7 (35.00)	0 (0.00)

Among the infection-related adverse reactions in group P, two were skin infections, which manifested as herpes zoster. The patients’ condition improved after treatment and they were not hospitalized. Among the infection-related adverse reactions in the P + CTX group, 1 case of pneumonia was considered severe and a serious adverse reaction, and after hospitalization, it was improved and the patient was discharged. There was 1 case of gastrointestinal bleeding in the P + CTX and P + MMF groups. Melena was the main clinical manifestation in the P + MMF group, without a significant hemoglobin drop, and the patients improved after outpatient treatment, such as strengthening the protection of the gastric mucosa. Patients in the P + CTX group had blood in the stool, accompanied by fatigue, pale complexion, and other hypovolemic manifestations, which were serious adverse reactions. They improved after treatment, with hemostasis, inhibition of gastric acid, and protection of the gastric mucosa. In addition, one case of transaminase was slightly elevated in the P + MMF group. These adverse reactions are considered to be related to the treatment drugs.

## Discussion

The severity of the renal pathological grade is independently associated with poor prognosis, and severe IgAN had a higher risk of progression to ESRD (Kim et al., 2012). Supportive care has limited efficacy in delaying the progression of severe IgAN, and some patients with severe IgAN who only receive supportive care progress rapidly to dialysis (Kim et al., 2012). However, guidelines for the treatment of severe IgAN are still unclear, therefore it is necessary to explore whether immunosuppressive therapy has a renoprotective effect on severe IgAN. This was a single-center retrospective study. The clinical follow-up data of patients with severe IgAN with Lee’s pathological grades IV–V in our center were collected, and the efficacy and adverse reactions of immunosuppressants were observed, hoping to provide evidence for the treatment of severe IgAN patients.

In this study, the baseline indicators (Scr, ALB, Cys C, BUN, HGB, and PCR) of the four groups of patients were not significantly different, suggesting that the four treatment groups had good comparability. One study (Towler et al., 2012) showed that PCR is a suitable substitute for 24-UPro, therefore this study used PCR to monitor the changes in the disease. In this study, at the 24th month of treatment, the PCR in three immunosuppressant groups was lower than that of the supportive care group. Moreover, the PCR in the four groups was significantly lower than that at baseline, and the ALB level was higher than that at baseline. This suggests that, despite the presence of diffuse glomerular hyperplasia, tubular atrophy, and renal interstitial inflammation, severe IgAN is still positively effected by immunosuppressive therapy. Most studies consider reducing uric acid to alleviate the inflammatory response in patients with chronic renal failure can promote the renal function (Yip et al., 2020). In this study, at the end of the 24-month treatment, the blood SUA levels of the patients in the P + MMF, P, and supportive care groups decreased compared with the baseline level after treatment, and the urine protein of the four groups decreased significantly compared with the baseline level. The decrease in SUA levels was considered to be associated with improved renal function and the use of uric acid-lowering drugs.

The 2020 KDIGO guidelines clearly recommend the long-term use of renin-angiotensin system inhibitors (RASI) for IgAN patients with urinary protein regardless of whether they have hypertension or not, and RASI drugs provide supportive care. In this study, after receiving supportive treatment for severe IgAN patients, although the PCR value decreased compared with the baseline value, the effective remission rate of urinary protein was only 29.4%, and renal function was not significantly improved compared with the baseline value. The possible reason is that, on the one hand, the patients in this study failed to use all RASI drugs, instead the center gave active immunosuppressive therapy to patients with severe IgAN, resulting in a small number of patients in the observed supportive care group. This represents bias. In the pathogenesis of IgAN, RASI drugs cannot improve the renal pathological damage caused by immune abnormalities.

Regarding the timing of immunosuppressant use, the KDIGO guidelines suggest that it should be used only for high-risk patients with urine protein greater than 1 g after 3 months of maximum supportive treatment. This study showed that the proportion of patients receiving prednisone therapy was 82.6%, whereas the proportion of patients receiving combined immunosuppressive drugs was 75.3%. A study reported that (Yonghua, 2012) adopted more aggressive immunosuppressive treatment regimens, among which prednisone combined with CTX had the highest proportion (88.5%), which is consistent with the treatment regimen in this study. This study was followed for 24 months. The results showed that the effective remission rates in the P, P + CTX, and P + MMF groups were 65%, 85.7%, and 61.6%, respectively, which were higher than the 29.4% in the supportive care group. Thus, immunosuppressive therapy is believed to be more effective than supportive therapy for reducing urinary protein levels.

The use of glucocorticoids is controversial. The results of the STOP-IgAN prospective study suggest that systemic corticosteroid therapy significantly reduces proteinuria, but does not slow disease progression in IgAN (Rauen et al., 2015). However, the risk of side effects caused by methylprednisolone treatment increased five times compared with placebo, and the study had to be stopped early (Lv et al., 2017). At the same time, the results of the NEFIGAN study suggest that budesonide targeted for ileal release can effectively reduce urinary protein in patients with IgAN (Fellström et al., 2017). Therefore, the latest guidelines recommend the use of glucocorticoids in patients with mild-to-moderate renal impairment and urinary proteins. The results of this study showed that in the early stage of treatment for patients with severe IgAN, the effective remission rate of prednisone therapy alone was 75%, which was 3.17 times that of the supportive treatment group, and with prolonged treatment time, prednisone could continue to reduce urinary protein, while the supportive treatment was effective. The remission rate decreased from 41.20% at 6th month to 29.40% at 24th month, suggesting that supportive treatment alone has limited efficacy in patients with severe IgAN. The study in our center is also similar to most studies, and the adverse reactions of prednisone therapy were also higher than those of the supportive care group. So, the pros and cons must be weighed in the use of prednisone.

Chen et al. (Chen et al., 2002) conducted the first RCT on MMF in the treatment of IgAN. The study included 62 IgAN patients with proteinuria greater than 2.0 g/d, serum creatinine less than 355 μmol/L, and Lee’s IV–V grades. Compared with prednisone therapy, MMF combined with prednisone therapy can reduce urinary protein levels and improve renal prognosis. Simultaneously, the results of a prospective study in Hong Kong showed that for IgAN patients with moderate renal insufficiency and proteinuria >1.8 g/d, the use of MMF resulted in a significant decrease in urinary protein and a higher 6-year renal survival rate (Tang et al., 2005). Not long ago, scholars reported that corticosteroids + MMF is an effective treatment in IgAN patients with a sustained decline in kidney function accompanied by persistent proteinuria and haematuria despite optimized conservative treatment (Huerta et al., 2022). However, foreign prospective studies have not found that MMF has a renoprotective effect on IgAN (Zheng et al., 2018). The inconsistency in research results in China and abroad suggests that genetic heterogeneity of different races has an impact on the pathogenesis of IgA. The results of this study showed that at the 18th month of follow-up, the effective remission rate in the P + MMF group was lower than that reported by Chen al. (Chen et al., 2002) (65.4% vs. 88%). Although the dose and duration of MMF drug treatment in the two groups of this study and those of Chen et al. (Chen et al., 2002) were similar, considering the possibility that it was associated with a higher proportion of patients with renal insufficiency in the MMF group enrolled in this study (68% vs. 19%). Therefore, the results of this study suggest that patients with severe IgAN with renal insufficiency are slightly less responsive to MMF therapy.

Cyclophosphamide (CTX) can significantly reduce urinary protein levels and reduce renal endpoint events (Fang et al., 2014). The guidelines also clearly state that CTX should only be used in patients with crescent-type IgAN with a rapid deterioration of renal function. However, for the vast majority of patients with non-crescent IgAN, especially those with progressive severe IgAN, the efficacy of CTX remains unclear. Studies have found certain similarities between the pathogenesis of IgAN and lupus nephritis, including selective activation of monocyte-derived and intrarenal cytokine systems, production of autoimmune IgA and IgG antibodies, and myeloid B-cell activation. Therefore, the combination of corticosteroids with CTX followed by MMF therapy in the classic treatment regimen of lupus nephritis may have potential benefits for patients with IgAN (Lafayette and Kelepouris, 2018). Rasche (Rasche et al., 2016) and others found that the median survival time of patients with CTX sequential MMF patients was 10.7 years, and the urinary protein level after treatment was significantly lower than the baseline, suggesting that sequential regimens can effectively reduce proteinuria and prolong renal survival time. In this study, at the 12th, 18th, and 24th months of treatment, the effective remission rate in the P + CTX group was higher than that in the supportive care group. The proportion of patients entering the endpoint in the P + CTX, P + MMF, and P groups (2.90%, 7.70%, and 10.00%, respectively) was significantly lower than that in the supportive care group (23.5%). Therefore, this study presumes that P + CTX can effectively reduce proteinuria in patients with severe IgAN early, thus reducing the occurrence of combined endpoint events and slowing the deterioration of renal function. Similarly, a recently literature report (Jia et al., 2022) that compared with corticosteroid + MMF therapy, corticosteroid + CTX therapy was more safety and possibly more effective. However, it is necessary to monitor the occurrence of adverse reactions, such as infection, during immunosuppressive therapy.

There are several limitations in this study. First, this is a single-center study with a limited number of cases, for these reasons, the conclusions drawn in this study should be further validated through large-scale studies with long follow-up periods. Second, this is a retrospective study, although we conducted baseline comparison on the enrolled patients (as shown in Table 1), and then conduct subsequent statistics and data analysis after there is no significant difference in baseline comparison, randomized prospective controlled trials are still needed to validate our findings in the further studies. Third, the follow-up period of this study was relatively short and the number of cases was small, thus long-term follow up of large-scale patients in clinical trials are still needed. Lastly, all participants in this study were Chinese in southwest part of China and the results of this study may have a relation with ethnic and regional factors.

## Conclusion

In conclusion, our center actively uses immunosuppressive therapy for patients with severe IgAN, and the combination of prednisone with cyclophosphamide followed by mycophenolate mofetil is the most commonly used regimen. Prednisone combined with cyclophosphamide in the treatment of severe IgAN have a high effective remission rate of urinary protein and a low incidence of end-point events, but attention should be paid to adverse drug reactions. Immunosuppressive therapy for severe IgA nephropathy can effectively reduce urinary protein levels, increase serum albumin levels, and protect renal function in the early stage of treatment.

## Data Availability

The raw data supporting the conclusion of this article will be made available by the authors, without undue reservation.
